# Analysis of the correlation between serum vitamin D and hypothalamic-pituitary-adrenal axis hormone levels in patients with post-traumatic stress disorder

**DOI:** 10.3389/fnins.2025.1622978

**Published:** 2025-09-29

**Authors:** Lei Ge, Weihua Xu, Wencong Liu, Panpan Cui, Lei Zhang, Hui Ju

**Affiliations:** ^1^Department of Emergency, People’s Hospital of Rizhao, Jining Medical University, Rizhao, Shandong, China; ^2^College of Clinical Medicine, Jining Medical University, Jining, Shandong, China; ^3^Department of ENT, People’s Hospital of Rizhao, Jining Medical University, Rizhao, Shandong, China

**Keywords:** post-traumatic stress disorder, vitamin D, hypothalamic-pituitary-adrenal axis, cortisol, adrenocorticotropic hormone, corticotropin-releasing hormone

## Abstract

**Objective:**

Post-Traumatic Stress Disorder (PTSD) is a psychological disorder triggered by extreme traumatic events. It is characterized by impaired cognitive function and neuroendocrine dysfunction, particularly dysregulation of the hypothalamic-pituitary-adrenal axis. In recent years, the role of vitamin D in neuroprotection and cognitive function has garnered increasing interest; however, its relationship with hypothalamic–pituitary–adrenal (HPA) axis hormone levels in patients with post-traumatic stress disorder (PTSD) remains poorly understood.

**Methods:**

This study aimed to investigate the correlation between serum vitamin D levels and HPA axis hormones in patients with PTSD. A total of 96 patients with severe trauma admitted to Rizhao People’s Hospital between March 2022 and December 2024 were enrolled and categorized into PTSD and non-PTSD groups according to diagnostic criteria. PTSD symptoms were evaluated using the PTSD Checklist–Civilian Version. Serum levels of 25-hydroxyvitamin D, corticotropin-releasing hormone, adrenocorticotropic hormone, and cortisol were measured. Spearman’s correlation analysis and receiver operating characteristic curves were employed to assess associations between vitamin D, HPA axis biomarkers, and PCL-C Scores.

**Results:**

The results showed that serum 25-hydroxyvitamin D levels were significantly lower in the PTSD group compared to the non-PTSD group (*P* < 0.001), while CRH and ACTH levels were significantly higher, and cortisol levels were significantly lower (*P* < 0.001). Spearman correlation analysis indicated that vitamin D levels were negatively correlated with CRH and ACTH levels and positively correlated with cortisol levels (*P* < 0.05). ROC curve analysis revealed that serum 25-hydroxyvitamin D levels have diagnostic potential for PTSD, with a cutoff value of 16.32 ng/mL, an AUC of 0.698, sensitivity of 86.2%, and specificity of 51.1%.

**Conclusion:**

This study demonstrated a correlation between serum vitamin D levels and HPA axis hormone levels in patients with PTSD, suggesting that vitamin D deficiency may be associated with HPA axis dysregulation in PTSD. These findings underscore a potential link between vitamin D deficiency and PTSD, warranting further investigation into the role of vitamin D in the disorder’s pathophysiology and its potential as a therapeutically modifiable factor.

## 1 Introduction

Post-traumatic stress disorder (PTSD) is a mental illness triggered by extreme traumatic events, leading to sustained changes in patients’ psychology, behavior, and cognitive functions. Patients with PTSD often suffer from significant psychological disorders and cognitive impairments, which severely affect their physical and mental health, increase the risk of adverse outcomes, and significantly reduce their quality of life (Jacquet-Smailovic et al., 2022; Mandagere et al., 2025; Padhi et al., 2024). In recent years, research has shown that the occurrence of PTSD is closely related to abnormalities in multiple neuroendocrine systems, especially the dysfunction of the hypothalamic-pituitary-adrenal (HPA) axis (Engel et al., 2023). The dysfunction of the HPA axis is considered a core neurobiological feature of PTSD. However, research findings regarding cortisol levels, the end-product of the HPA axis, have been complex and sometimes contradictory. While a substantial body of literature supports the model of heightened negative feedback inhibition, leading to lower baseline cortisol levels and glucocorticoid receptor supersensitivity in PTSD (Speer et al., 2019; Yehuda and LeDoux, 2007), this is not a universal finding. Other studies have reported elevated cortisol levels or no significant differences compared to controls in certain patient subgroups or under specific conditions (Al-Kufaishi and Al-Musawi, 2024). This heterogeneity may be attributed to factors such as trauma type, chronicity of PTSD, comorbidity with other disorders, and timing of sample collection. Despite these discrepancies, the majority of the literature emphasizes the crucial role of HPA axis dysregulation in the development and maintenance of PTSD (Dunlop and Wong, 2019). On the other hand, vitamin D, an essential nutrient, not only participates in calcium and phosphorus metabolism but also influences the regulation of the nervous system and immune function. Studies have indicated that vitamin D deficiency may be associated with the development of various mental disorders and plays an important role in neuroprotection and cognitive function regulation (Cui et al., 2021; da Silva et al., 2022; Zhang et al., 2019). However, there have been few studies on the correlation between serum vitamin D levels and HPA axis hormone levels in patients with PTSD (Wierzbicka et al., 2016). This study aims to explore the correlation between serum vitamin D levels and HPA axis hormone levels in patients with PTSD, to further uncover the pathogenesis of PTSD, and to provide new ideas and evidence for clinical intervention.

## 2 Materials and methods

### 2.1 Case selection criteria

Inclusion criteria: (1) Patients with severe trauma (ISS score > 16) (Mlaver et al., 2024); (2) PTSD patients diagnosed by a psychiatrist according to the Chinese Classification and Diagnostic Criteria of Mental Disorders, 3rd edition (Miao et al., 2018); (3) No history of other mental illnesses; (4) Complete clinical data; (5) No significant organ diseases, metabolic syndrome, diabetes, gout, hyperthyroidism, or blood disorders.

Exclusion criteria: (1) History of drug abuse affecting sleep, mood, cognition, or immunity; (2) Other major illnesses causing anxiety, panic, or PTSD symptoms requiring urgent intervention; (3) Pregnancy or breastfeeding; (4) The patient has used medications within the past 3 months that may significantly affect HPA-axis hormones or vitamin D metabolism, including but not limited to systemic corticosteroids, vitamin D or calcium supplements, and antidepressant drugs.

All participants were evaluated at 6 weeks following the traumatic injury. At this follow-up, venous blood was drawn for biochemical analysis, and PTSD symptoms were assessed using the PCL-C by physicians in the Memory Clinic.

### 2.2 General data

From March 2022 to December 2024, 96 severe trauma patients (ISS score > 16) were selected from Rizhao People’s Hospital. Using the PTSD Checklist Civilian Version, patients were divided into PTSD and non-PTSD groups. No significant differences were found in age, gender, body mass index (BMI), education, smoking, alcohol use, or ISS scores between the two groups (*P* > 0.05), Table 1. The PTSD incidence was 39.58%. The study was approved by the ethics committee of Rizhao People’s Hospital (approval No.: MR-95-03) and registered at the China Clinical Trial Registration Center. All participants provided informed consent.

**TABLE 1:** Comparison of general information between the two groups of patients.

Group	Age (years)	Gender (male/female)	BMI (kg/m^2^)	Education (years)	Smoking history (yes/no)	Alcohol history (yes/no)	ISS score
PTSD group (*n* = 38)	42.3 ± 5.4	23/15	21.0 ± 0.3	12.5 ± 2.1	18/20	17/21	24.5 ± 5.2
Non-PTSD group (*n* = 58)	42.6 ± 5.8	39/19	21.1 ± 0.4	13.2 ± 2.4	28/30	27/31	25.1 ± 5.5
t/χ^2^	0.345	0.123	0.876	1.234	0.045	0.012	0.678
*P*	0.731	0.726	0.382	0.221	0.831	0.912	0.5

### 2.3 Research methods

#### 2.3.1 Assessment of PTSD symptoms

The PTSD Checklist Civilian Version (PCL-C) was used to evaluate PTSD symptoms across three dimensions: re-experiencing, avoidance/numbing, and increased arousal. Scores of 17–37 indicate no significant PTSD symptoms, 38–49 suggest some symptoms, and 50–85 indicate marked symptoms (Mureşanu et al., 2022). Participants with a PCL-C total score <38 were classified as the non-PTSD group, while those with a score ≥38 were classified as the PTSD group.

#### 2.3.2 Detection of serum 25-hydroxyvitamin D, corticotropin-releasing hormone, adrenocorticotropic hormone, and serum cortisol

All blood samples were collected in the early morning (between 7:00 and 9:00 AM) after an overnight fast to control for the pronounced diurnal variation in cortisol levels. The blood samples were immediately transported to the central laboratory of our hospital for the detection of 25-hydroxyvitamin D, corticotropin-releasing hormone (CRH), adrenocorticotropic hormone (ACTH), and serum cortisol (COR). The levels of 25-hydroxyvitamin D, CRH, and ACTH were measured using ELISA method. COR was measured using CLIA. All reagent kits were provided by Shanghai Enzyme-Linked Biotechnology Co., Ltd.

### 2.4 Statistical analysis

Data were analyzed using SPSS 25.0. Shapiro-Wilk test assessed normality. Normally distributed data were expressed as mean ± SD, and non-normally distributed data as median (interquartile range). Independent samples *t*-test compared normally distributed data between groups, and non-parametric tests compared non-normally distributed data. Bivariate correlations were analyzed using Spearman’s test. ROC curves evaluated the diagnostic efficacy and cutoffs for serum 25-hydroxyvitamin D, CRH, ACTH, and COR in PTSD post-trauma. Post-cutoff grouping compared emotional differences using chi-square tests. Statistical significance was set at *P* < 0.05.

## 3 Results

### 3.1 Basic demographic characteristics of the study participants

A total of 96 patients with severe trauma were included, with a mean age of 43.23 ± 5.5 years. Among the participants, 62 (64.6%) were male and 34 (35.4%) were female. In terms of marital status, 21 (21.9%) were unmarried while 75 (78.1%) were married. Regarding lifestyle factors, 46 (47.9%) participants reported smoking and 44 (45.8%) reported alcohol consumption.

Post-traumatic stress disorder was diagnosed in 38 patients, corresponding to an incidence rate of 39.6%. Using the PTSD Checklist-Civilian Version (PCL-C) score as the dependent variable, and gender, marital status, smoking, and drinking as independent variables, univariate analysis showed that none of these factors were statistically significant (*P* > 0.05).

### 3.2 Comparison of hormone levels between PTSD and non-PTSD groups after severe trauma

In patients with severe trauma, the levels of 25-hydroxyvitamin D, CRH, ACTH, and COR were significantly different between the PTSD and non-PTSD groups. In the PTSD Group, the levels of 25-Hydroxyvitamin D were 16.62 ± 2.88 ng/mL, CRH was 127.14 ± 13.45 ng/mL, ACTH was 40.03 ± 4.92 pg/mL, and COR was 174.55 ± 23.58 μg/dL. In the Non-PTSD Group, the levels of 25-Hydroxyvitamin D were 18.54 ± 2.11 ng/mL, CRH was 107.14 ± 11.30 ng/mL, ACTH was 33.37 ± 4.61 pg/mL, and COR was 200.03 ± 20.36 μg/dL. There were significant differences in all these parameters between the two groups, which were statistically significant (*p* < .001) see Table 2.

**TABLE 2:** Comparison of hormone levels in two groups.

Group	25-hydroxyvitamin D (ng/mL)	CRH (pg/mL)	ACTH (pg/mL)	COR (μg/dL)	PCL-C scores
PTSD group (*n* = 38)	16.62 ± 2.88	127.14 ± 13.45	40.03 ± 4.92	174.55 ± 23.58	47.81 ± 4.03
Non-PTSD group (*n* = 58)	18.54 ± 2.11	107.14 ± 11.30	33.37 ± 4.61	200.03 ± 20.36	29.58 ± 2.46
t (z) value	−3.52	−5.60	−5.18	−5.04	24.94
*P*-value	<0.001	<0.001	0.001	<0.001	<0.001

In the PTSD group, vitamin D showed a strong negative correlation with CRH (*r* = −0.475, *p* < 0.01) and ACTH (*r* = −0.466, *p* < 0.01), and a strong positive correlation with COR (*r* = 0.559, *p* < 0.01). In the non-PTSD group, vitamin D was only weakly negatively correlated with ACTH (*r* = −0.285, *p* < 0.05), and no significant correlations were found with CRH or COR see Tables 3, 4.

**TABLE 3:** Correlation analysis of hormone levels in PTSD group.

Project	Vitamin D	CRH	ACTH	COR
Vitamin D	1			
CRH	−0.475^**^	1		
ACTH	−0.466^**^	0.472^**^	1	
COR	0.559^**^	−0.493^**^	−0.340*	1

^**^p < 0.01,

*p < 0.05.

**TABLE 4:** Correlation analysis of hormone levels in non-PTSD group.

Project	Vitamin D	CRH	ACTH	COR
Vitamin D	1			
CRH	−0.014	1		
ACTH	−0.285*	0.204	1	
COR	0.229	−0.271*	−0.2	1

**p < 0.01.

### 3.3 Correlation analysis of vitamin D, pituitary axis, and PCL-C scores

Spearman correlation analysis across the entire cohort of trauma patients (*n* = 96) showed that vitamin D levels were significantly correlated with the pituitary axis hormones and PCL-C Scores (*P* < 0.05), as detailed in Table 5.

**TABLE 5:** Correlation analysis of vitamin D, pituitary axis, and PCL-C scores.

Project	Vitamin D	CRH	ACTH	COR	PCL-C scores
Vitamin D	1				
CRH	−0.312**	1			
ACTH	−0.239*	0.395**	1		
COR	0.255*	−0.356**	−0.226*	1	
PCL-C scores	−0.380**	0.591**	0.550**	−0.443**	1

**p < 0.01,

*p < 0.05.

**FIGURE 1:**
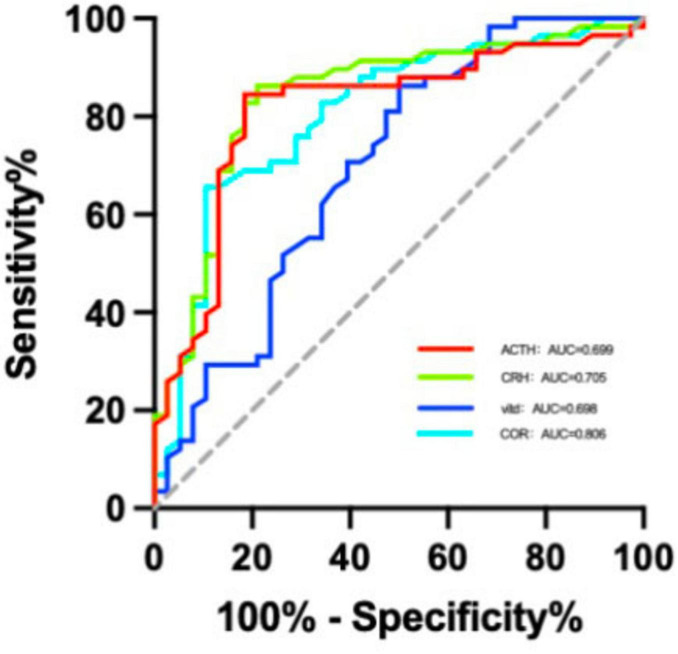
ROC curves for diagnosing PTSD using HPA axis hormones and vitamin D.

The ROC curve analysis revealed that in severe trauma patients, the optimal cutoff value for vitamin D in predicting PTSD was 16.32 pg/mg, with an AUC of 0.698 (95% CI: 0.587, 0.81), sensitivity of 86.2%, and specificity of 51.1%. For CRH, the cutoff was 107.675 pg/mg, AUC 0.705 (95% CI: 0.075, 0.246), sensitivity 78.9%, and specificity 86.2%. For ACTH, the cutoff was 36.54 pg/mg, AUC 0.699 (95% CI: 0.094, 0.277), sensitivity 81.6%, and specificity 84.5%. For COR, the cutoff was 195.655, AUC 0.806 (95% CI: 0.714, 0.898), sensitivity 65.5%, and specificity 89.5%. After grouping participants based on these cutoffs, chi-square tests showed significant differences in emotional outcomes between groups (*P* < 0.05) see Figure 1 and Tables 6, 7.

**TABLE 6:** Cut-off values of pituitary axis hormones and vitamin D for PTSD.

		95% CI					
Project	AUC	Lower limit	Upper limit	*P*-value	Cut-off value	Sensitivity	Specificity
Vitamin D	0.698	0.587	0.81	*p* < 0.001	16.32	0.862	0.511
CRH	0.705	0.075	0.246	*p* < 0.001	107.675	0.789	0.862
ACTH	0.699	0.094	0.277	*p* < 0.001	36.54	0.816	0.845
COR	0.806	0.714	0.898	*p* < 0.001	195.655	0.655	0.895

**TABLE 7:** Comparison of PTSD among groups after stratification by cut-off values (*n*).

Group	Vitamin D		CRH		ACTH		COR	
Cut-off value	<16.32	≥16.32	<107.675	≥107.675	<36.54	≥36.54	<195.655	≥195.655
PTSD	19	19	8	30	7	31	34	4
Non-PTSD	8	50	50	8	49	9	20	38
Chi-square value	14.88		40.75		41.22		28.21	
*P*-value	*p* < 0.001		*p* < 0.001		*p* < 0.001		*p* < 0.001	

## 4 Discussion

Post-traumatic stress disorder (PTSD) is a delayed stress response following abnormal physical or psychological trauma, marked by heightened vigilance, sustained mental exhaustion, and persistent anxiety. After severe trauma, patients often struggle with self-acceptance due to negative psychological experiences such as trauma-related stress, social detachment, physical changes, and functional disabilities, making them prone to PTSD (Maercker et al., 2022). In clinical practice, the focus is mostly on alleviating patients’ physical pain and restoring physiological functions, with less attention paid to their subjective experiences and psychological recovery. This lack of focus on psychological aspects can lead to poor long-term outcomes and worsening of PTSD symptoms (Williamson et al., 2021). Therefore, exploring the pathogenesis of PTSD in severe trauma patients and identifying prevention and intervention targets is crucial. In this study, 38 out of 96 severe trauma patients developed PTSD (39.58%), aligning with previous research findings.

The HPA axis, a key endocrine regulatory system, plays a vital role in stress response, energy metabolism, and immune reactions. Normally, when faced with stress, the hypothalamus releases CRF, prompting the pituitary to secrete ACTH, which then stimulates cortisol production from the adrenal glands. These stress hormones, through a negative feedback loop, modulate HPA axis activity, with ACTH and cortisol inhibiting the hypothalamus and pituitary to prevent excessive stress hormone production (Herman et al., 2016). However, in PTSD patients, HPA axis dysfunction manifests as elevated CRF and low cortisol levels. Cortisol, crucial for regulating stress responses, enhances the body’s resistance and adaptability (Dunlop and Wong, 2019). This study found heightened CRF and lowered cortisol in PTSD patients, indicating HPA axis dysregulation. Reduced cortisol can lead to central nervous system stress dysregulation, causing symptoms like irritability, excessive fear, heightened sensitivity to trauma-related cues, and flashbacks (Hagedorn et al., 2021; Trevino et al., 2022; Wichmann et al., 2017). It also boosts sympathetic nervous system activity and memory consolidation, deepening traumatic memories (Ballhausen et al., 2019; Morris et al., 2016). Additionally, abnormal cortisol circadian rhythms in PTSD patients further underscore the role of HPA axis dysfunction in the disorder’s development (Sterina et al., 2022).

Vitamin D, a lipophilic sterol derivative, primarily exists in two major forms: ergocalciferol (vitamin D2) and cholecalciferol (vitamin D3). Vitamin D3 is synthesized in the skin through ultraviolet-induced photochemical conversion of 7-dehydrocholesterol and can also be obtained from animal-derived dietary sources. In contrast, vitamin D2 is derived from plant-based foods. Upon entering the circulation, vitamin D binds to vitamin D-binding protein and is transported via the bloodstream to various target tissues. Its biological effects are mediated through binding to the vitamin D receptor, a nuclear receptor that regulates gene expression. Accumulating evidence from recent studies underscores an association between vitamin D deficiency and psychiatric disorders, highlighting its significance in neuroprotective mechanisms and the modulation of cognitive function (Cui et al., 2021; da Silva et al., 2022). In this research, the PTSD group had significantly lower serum 25-hydroxyvitamin D levels than the non-PTSD group. The significant correlations among CRH, ACTH, and cortisol in the PTSD group further support the notion of HPA axis hyperactivity and impaired negative feedback in PTSD pathophysiology. In contrast, the weaker and fewer correlations in the non-PTSD group suggest a more tightly regulated and homeostatic HPA axis. Similarly, ROC analysis demonstrated a modest yet statistically significant association between vitamin D levels and PTSD status (AUC = 0.698, *p* < 0.001). These findings may be attributed to the analysis being conducted in a combined cohort of both PTSD and non-PTSD patients. The modest correlation values suggest that the relationship between vitamin D and HPA axis hormones could be influenced by other unmeasured factors within this heterogeneous trauma population, reflecting the complex multifactorial pathophysiology of PTSD. Therefore, further extensive basic research is warranted to elucidate the specific pathophysiological mechanisms underlying this relationship. Additionally, future studies with larger sample sizes are necessary to explore these associations across different subgroups.

This study observed a correlation between serum vitamin D levels and hormones of the HPA axis. Vitamin D may exert neuroprotective and HPA-regulatory effects through specific molecular mechanisms. The active form of vitamin D, calcitriol, binds to the vitamin D receptor (VDR), which is widely expressed in stress-responsive brain regions such as the hypothalamus, hippocampus, and prefrontal cortex (Al-Kuraishy et al., 2024). Activation of VDR signaling can modulate the transcription of genes involved in neuroendocrine regulation and inflammatory responses (He et al., 2025). For instance, vitamin D has been shown to downregulate the expression of CRH and pro-inflammatory cytokines such as IL-6 and TNF-α, which are typically elevated in PTSD and contribute to HPA axis hyperactivity (Abiri et al., 2022; Eyles et al., 2006; Mohamed et al., 2018). Furthermore, vitamin D enhances the expression of anti-inflammatory cytokines and neurotrophic factors such as BDNF, which support neuronal survival and synaptic plasticity (Cui et al., 2020). Together, these mechanisms suggest that vitamin D may alleviate HPA axis dysregulation–and thereby mitigate characteristic pathological stress responses in PTSD–not only through direct hormonal feedback but also via immunomodulatory and neurotrophic pathways.

It is also noteworthy that the chronic stress inherent in PTSD and HPA axis dysregulation may in turn affect vitamin D metabolism (Karin et al., 2020; Luthra et al., 2022). Glucocorticoids can influence the expression of enzymes involved in vitamin D metabolism, while behavioral changes associated with PTSD–such as social withdrawal and reduced outdoor activity–may lead to decreased sunlight exposure, which is the primary source of vitamin D (Asch et al., 2021; Penckofer et al., 2010). Therefore, the observed low vitamin D levels may be both a contributing factor and a consequence of PTSD-related pathology, warranting further in-depth investigation.

Our study has several limitations that must be acknowledged. First, as mentioned earlier, the cross-sectional design limits causal interpretation. Second, we did not account for several potential confounding factors influencing vitamin D status, such as seasonal variations in sunlight exposure, dietary intake, supplement use, or BMI. Although no significant difference in BMI was observed between the two groups, other unmeasured factors may have affected both vitamin D levels and PTSD risk. Future longitudinal or interventional studies should strive to control for these variables to clarify the nature of this relationship. Third, the AUC for vitamin D in predicting PTSD was 0.698, indicating only moderate discriminatory power. Therefore, although vitamin D shows promise as a potential biomarker, it should not be considered as a standalone diagnostic tool, but rather be incorporated as one component within a broader risk assessment model.

In summary, this study demonstrates a significant correlation between serum vitamin D levels and HPA axis hormone levels in patients with PTSD. These findings reveal potential physiological underpinnings of PTSD and suggest that vitamin D status, as a modifiable factor, warrants further investigation in relation to the disorder. Future longitudinal and interventional studies will be essential to determine the direction of causality and to explore whether vitamin D supplementation may play a beneficial role in the management of PTSD.

## Data Availability

The raw data supporting the conclusions of this article will be made available by the authors, without undue reservation.
